# A young woman presented with massive pulmonary embolism with inferior vena cava thrombus as a complication of nephrotic syndrome: a case report

**DOI:** 10.1186/s12245-021-00369-2

**Published:** 2021-08-23

**Authors:** Mohamed Osman Omar Jeele, Rukia Omar Barei Addow, Mohamed Farah Yusuf Mohamud

**Affiliations:** 1 Mogadishu Somali Turkish Training and Research Hospital, Mogadishu, Somalia; 2 Jazeera University Hospital, Mogadishu, Somalia

**Keywords:** Nephrotic syndrome, Thromboembolism, Inferior vena cava thrombosis, Pulmonary embolism

## Abstract

Nephrotic syndrome (NS) was first described in 1827 as the presence of proteinuria of ≥ 3.5 g/24 h, hypoalbuminemia < 3.0 g/dl, peripheral edema, hyperlipidemia, lipiduria, and increased thrombotic risk. Nephrotic syndrome has an incidence of three cases per 100,000 each year in adults. Nephrotic syndrome also has serious complications due to hypercoagulable state in both various venous and arteries which could lead thromboembolic events. The pathophysiology of hypercoagulability in the nephrotic syndrome is due to an imbalance of prothrombotic and antithrombotic factors, as well as impaired thrombolytic activities.

Here, we will present a 19-year-old woman who presented to the emergency department complaining of chest pain and shortness of breath for 3 days. The patient was quickly diagnosed with pulmonary embolism and inferior vena cava thrombosis as a complication of nephrotic syndrome, allowing prompt initiation of anticoagulant therapy. After 2 weeks of admission, the patient’s condition resolved, her laboratory results returned to almost normal and the patient was discharged with oral prednisolone, coumadin, atorvastatin, and ramipril. We aim to determine which is the likely cause of pulmonary embolism in patients with nephrotic syndrome.

## Introduction

Nephrotic syndrome (NS) was first described in 1827 as the presence of proteinuria of ≥ 3.5 g/24 h, hypoalbuminemia < 3.0 g/dl, peripheral edema, hyperlipidemia, lipiduria, and increased thrombotic risk [1, 2]. Nephrotic syndrome has an incidence of three cases per 100,000 each year in adults [3]. Thromboembolism, including pulmonary embolism, deep venous thrombosis (DVT), renal vein thrombosis, and inferior vena cava thrombosis has been reported as a life-threatening complication of nephrotic syndrome patients [4]. Enhanced platelet aggregation and loss of anticoagulant proteins including antithrombin III through renal are thought to cause the excessive thrombotic risk in patients with nephrotic syndrome [4, 5]. Here, we present a case of an unusual combination of multiple venous thromboses as the presentation of nephrotic syndrome.

## Case report

A 19-year-old woman, previously healthy, nonsmoker, non-diabetic came to the emergency department with a complaint of chest pain and shortness of breath for 3 days.

She never had a similar incident before. She does not have any history of drug use including oral contraceptive drugs. On examination, the patient looked ill, anxious, tachyapneic, and edematous. On vital signs, her respiratory rate was 23 breath/min, blood pressure 110/70 mm/Hg, pulse 112 bpm, and oxygen saturation was 90%. Head and neck examination were unremarkable. Tachycardia was the only prominent sign of cardiovascular findings. The respiratory evaluation revealed diminished breath sounds in both lungs. Grade 2+ lower limb edema noted during the examination while other systems examination were unremarkable. Laboratory investigations including complete blood count and serum electrolytes revealed normal range. Regarding the liver function test, AST was 35 U/L, ALT was 16 U/L, total protein was 4 g/dl, and Albumin was 1.9 g/dl. Total cholesterol was 450 mg/dl and lactate dehydrogenase was 639 U/l. HBV, HCV, HIV, and RF were all in the normal range or negative. The D-dimer (> 4 μg /ml) and 24-h urinary protein excretion (10 g) were positive. As there had no obvious predisposing factor for PE, tests for the etiology of the thrombophilic state were ordered. While waiting the results in the emergency, oxygen therapy with high flow nasal cannula was started along with subcutaneous low molecular weight heparin.

**Fig. 1:**
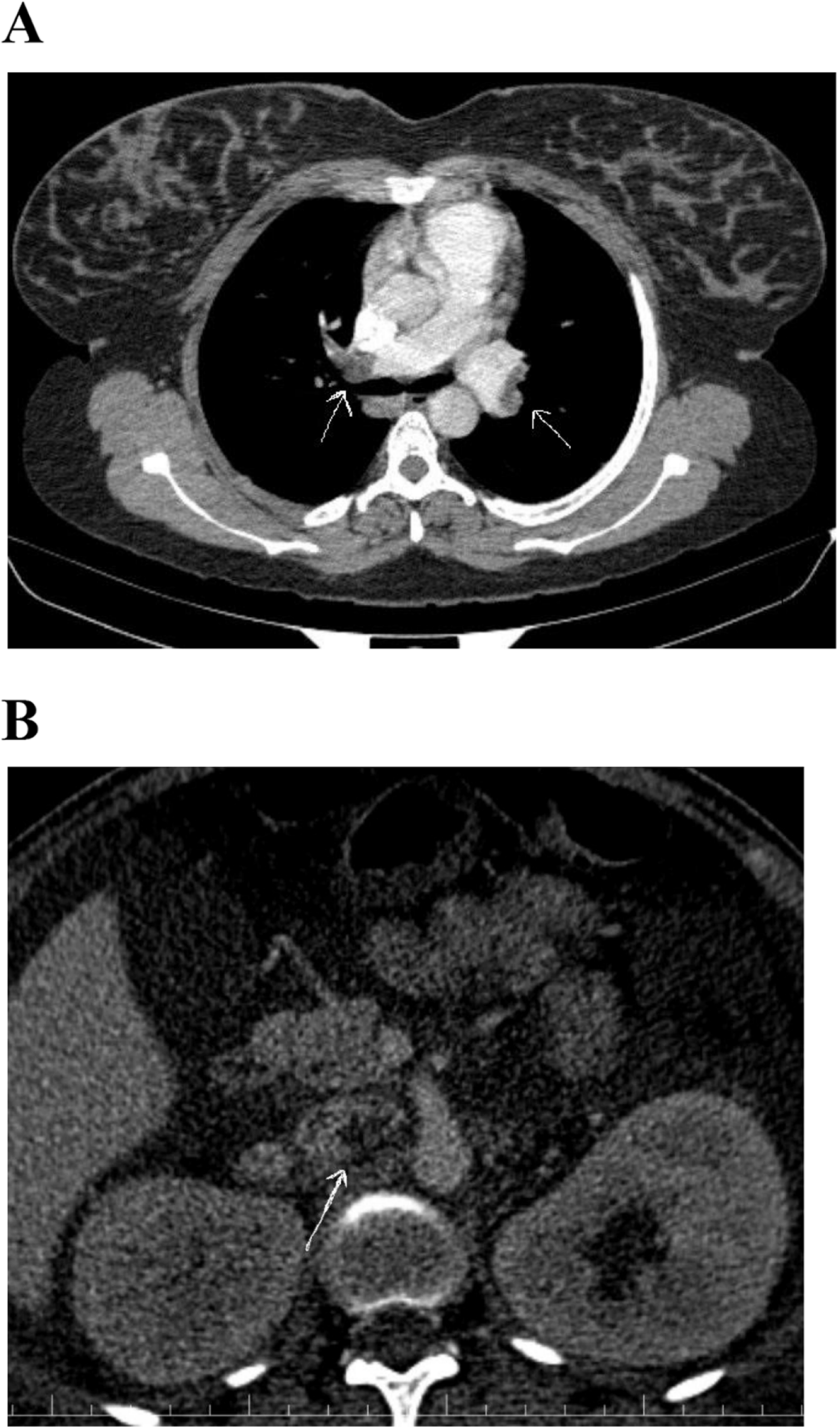
**A** Computed tomography pulmonary angiography revealed intraluminal filling defects representing emboli in the right and also left pulmonary arteries (PE). **B** An abdominal CT scan showed thrombus in the inferior vena cava

Chest X-ray and electrocardiograms were unremarkable. Abdominal ultrasonography detected mild ascites. Computed tomography (CT) angiography of the chest and abdomen revealed thrombus at both right and left pulmonary arteries and inferior vena cava (Fig. 1A and B). The patient was admitted for nephrotic syndrome complicated with pulmonary embolism and inferior vena cava thrombosis.

**Fig. 2:**
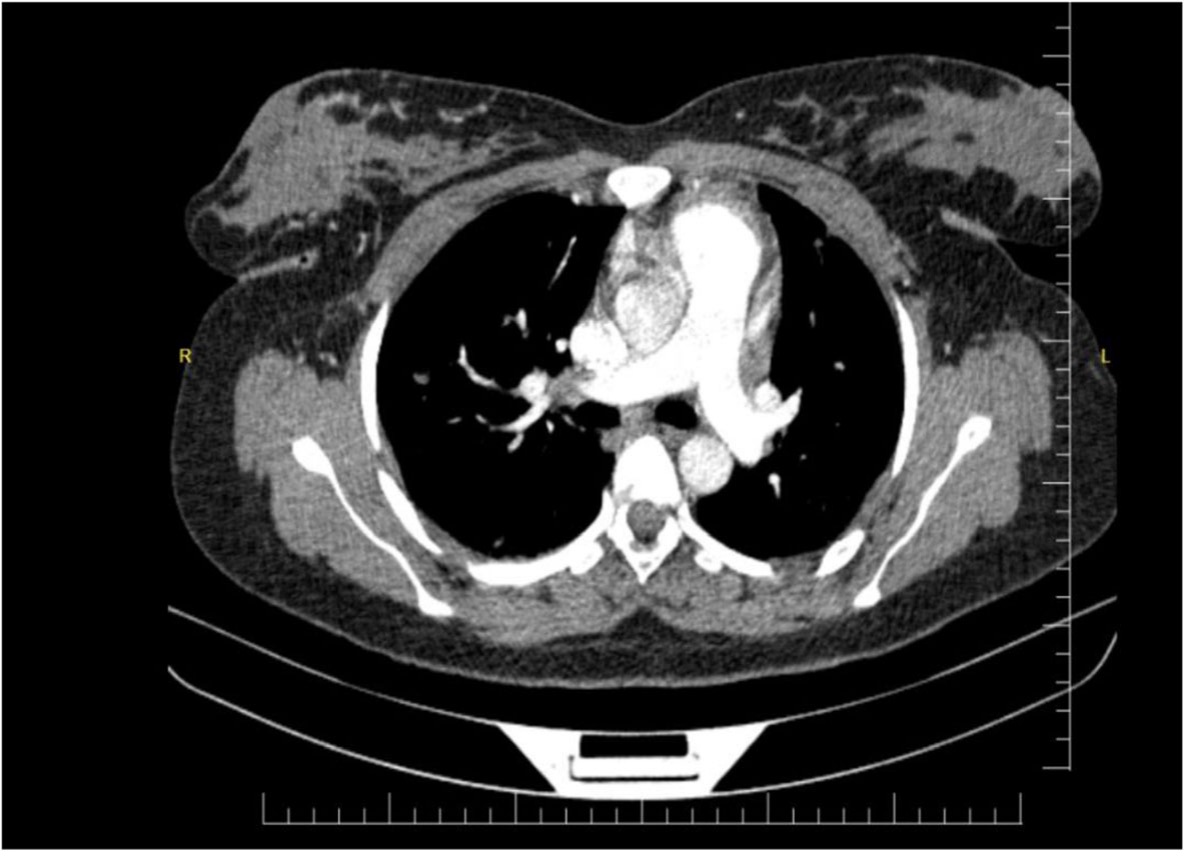
Computed tomography pulmonary angiography revealed normal pulmonary vessels with no thrombus or emboli

The patient was started on heparin, ramipril, methylprednisolone, and atorvastatin. Two days later, coumadin tablet was added. After 2 weeks of hospitalization, the patient symptoms resolved, lower limb edema decreased, proteinuria was not detected, and albumin returned to the normal range. The patient was discharged with oral prednisolone, coumadin, atorvastatin, and ramipril. Close follow-up was recommended for the patient. The patient’s 6-month follow-up revealed no complaints and no thrombus on the repeated chest CT angiography (Fig. 2).

## Discussion

Patients with nephrotic syndrome carry a high risk of both venous and arterial thrombosis [4]. Pulmonary embolism, deep venous thrombosis (DVT), renal vein thrombosis, and inferior vena cava thrombosis are reported as life-threatening complications of nephrotic syndrome [4, 5].

The etiology of NS is divided into primary, which includes focal segmental glomerulosclerosis (FSGS), membranous nephropathy (MN), and minimal change disease (MCD), and secondary to systemic diseases which included diabetes mellitus, systemic lupus erythematosus, multiple myeloma, amyloidosis, and infections [6]. The pathophysiology of hypercoagulability in the nephrotic syndrome is due to imbalances of prothrombotic and antithrombotic factors, impaired thrombolytic activity, and other important contributing factors such as intravascular volume depletion, the use of diuretics, immobilization, and procoagulant diatheses (such as protein C and protein S deficiencies) [7].

Hull RP et al. reported deep venous thrombosis (DVT) at the lower limbs as the most common complication of nephrotic syndrome, but also noted thrombosis at renal and pulmonary vessels [6]. Our case initially presented with pulmonary embolism as a complication of NS with no previous history of NS which is uncommon.

Similar to the presented case, Peces R et al. [8] reported a 42-year-old male that presented with multiple venous thromboses and pulmonary artery embolism as a complication of nephrotic syndrome. In our case, pulmonary embolism and inferior vena cava thrombosis were complications of nephrotic syndrome while persistent adequate renal blood flow prevented renal infarction.

## Conclusion

Nephrotic syndrome is a risk factor for venous thromboembolism due to increased renal loss of anticoagulant proteins included antithrombin III and increased production of pro-thrombotic factors by the liver. The possible occurrence of PE in a young person with nephrotic syndrome should not be missed. Early diagnosis and management of nephrotic syndrome may prevent the occurrence of venous thromboembolism (VTE).

## Data Availability

The data is available from the corresponding author and can be accessed if requested.

## Abbreviations

CT: Computed tomography
DVT: Deep venous thrombosis
FSGS: Focal segmental glomerulosclerosis
HBV: Hepatitis B virus
HCV: Hepatitis C virus
HIV: Human immunodeficiency virus
MCD: Minimal change disease
MN: Membranous nephropathy
NS: Nephrotic syndrome
RF: Rheumatoid factor
VTE: Venous thromboembolism

## References

[1] Bennett WM (1975). Renal vein thrombosis in nephrotic syndrome. Ann Intern Med..

[2] Cameron JS, Hicks J (2002). The origins and development of the concept of a nephrotic syndrome'. Am J Nephrol..

[3] Llach F (1984). Thromboembolic complications in nephrotic syndrome: coagulation abnormalities, renal vein thrombosis, and other conditions. Postgraduate Med..

[4] Kerlin BA, Ayoob R, Smoyer WE (2012). Epidemiology and pathophysiology of nephrotic syndrome-associated thromboembolic disease. Clin J Am Soc Nephrol..

[5] Llach F (1985). Hypercoagulability, renal vein thrombosis, and other thrombotic complications of nephrotic syndrome. Kidney Int..

[6] Hull RP, Goldsmith DJ (2008). Nephrotic syndrome in adults. Bmj..

[7] Singhal R, Brimble KS (2006). Thromboembolic complications in the nephrotic syndrome: pathophysiology and clinical management. Thrombosis Res..

[8] Peces R, Pobes A, Rodriguez M, Navascués RA, Ortega F, Alvarez-Grande J (1999). Multiple venous thrombosis and massive pulmonary artery thrombus as the presenting features of steroid-responsive nephrotic syndrome. Nephrol Dialysis Transplantation.

